# Mental Health Matters: A Multi‐Institution Study on the Mental Health of Undergraduate Dental Students

**DOI:** 10.1111/eje.70065

**Published:** 2025-11-26

**Authors:** Sadeq Ali Al‐Maweri, Kamran Ali, Daniel Zahra, Zohaib Khurshid, Alhanouf A. Alkhuraiji, Nourhan M. Aly, Anas Shamala, Abeer Abdulkareem Almashraqi, Alaa Daud

**Affiliations:** ^1^ Department of Preclinical Oral Sciences College of Dental Medicine, QU Health, Qatar University Doha Qatar; ^2^ College of Dental Medicine QU Health, Qatar University Doha Qatar; ^3^ Assessment and Psychometrics School of Psychology, University of Plymouth Plymouth UK; ^4^ Department of Prosthodontics and Dental Implantology College of Dentistry, King Faisal University Hofuf Saudi Arabia; ^5^ Center of Excellence for Regenerative Dentistry, Department of Anatomy Faculty of Dentistry, Chulalongkorn University Bangkok Thailand; ^6^ Department of Preventive Dental Sciences College of Dentistry, Majmaah University Al‐Majmaah Saudi Arabia; ^7^ Department of Pediatric Dentistry and Dental Public Health Alexandria University Alexandria Egypt; ^8^ Department of Preventive and Biomedical Science, Faculty of Dentistry UST Aden Yemen

**Keywords:** anxiety, dental students, depression, education, mental health, stress

## Abstract

**Purpose:**

This study aims to explore the prevalence of mental health issues among dental students in the Arabic‐speaking countries and identify the contributing factors.

**Methods:**

A cross‐sectional survey was conducted among dental students at multiple dental institutions from four Arabic‐speaking countries namely, Saudi Arabia, United Arab Emirates, Yemen, and Egypt. The survey questionnaire consisted of five sections: Consent to participate; Demographics of the participants; The Depression Anxiety Stress Scale (DASS‐21); the Patient Health Questionnaire (PHQ‐9); and open‐ended items.

**Results:**

Of the 508 responses received, 429 provided complete PHQ‐9 and DASS‐21 scales, forming the basis of the analyses. Results indicated a high prevalence of depression, anxiety, and stress among dental students, with 34.27% self‐reporting moderate depression and 9.86% severe depression. Additionally, significant gender differences were observed, with female students reporting higher levels of mental health symptoms for both DASS‐21 and PHQ‐9. The study also highlights the lack of significant association between year of study and severity of mental health symptoms, suggesting that mental health challenges are pervasive across all academic years.

**Conclusion:**

These findings underscore the need for targeted interventions to support the mental well‐being of dental students, with particular attention to academic stress, gender differences, and cultural considerations. By addressing these challenges, dental education programs can foster a healthier, more supportive learning environment for students.

## 1 Introduction

Mental health is widely recognised as a crucial component of overall well‐being. The World Health Organization (WHO) emphasised this in 1948 by defining health as “*A state of complete physical, mental, and social well‐being and not merely the absence of disease or infirmity*” [1]. The well‐being of individuals encompasses more than just the absence of illness; it also involves cultivating mental resilience, emotional balance, and fostering social connections. Globally, mental health disorders are a leading cause of disability and according to WHO, depression is the single largest contributor to global disability, affecting over 264 million people globally [2].

The psychological well‐being of students is a topic of growing interest and published research shows a high prevalence of poor psychological health in college [3] and university students [4]. A recent study showed that approximately one in three college students worldwide experiences significant symptoms of psychological issues, such as depression, anxiety, and suicidal ideation [5]. The psychological well‐being of healthcare students may be influenced by a multitude of factors including assessments, fear of failure, academic overload, strained faculty‐student relationships, financial burdens, and social isolation, among others [6, 7]. Moreover, in developing countries with limited resources and healthcare infrastructure, the increased prevalence of these stressors exacerbates mental health challenges among students [8].

A strong association of academic workload with anxiety and depression has been reported among medical students [9]. Similar findings are reported among dental students as the stress and anxiety associated with academic workload and assessments affect their physical health and psycho‐emotional well‐being adversely [10]. Undergraduate dental education requires dental students to perform invasive and irreversible procedures which can be a significant source of stress and anxiety [11]. A recent study on undergraduate dental students showed that 48.21% of students were screened positively for moderate to extremely severe depression, 49.30% for moderate to extremely severe anxiety and 30.36% of participants showed features of moderate to extremely severe stress [12].

Dental education is universally demanding, but in many Arabic‐speaking countries, students often face additional stressors such as high academic expectations, societal pressure, limited access to mental health resources, and stigma associated with psychological distress [13, 14]. The existing research on the mental well‐being of dental students has largely focused on Western or non‐Arabic populations, resulting in a critical gap in the literature regarding the experiences and support needs of Arabic‐speaking dental students. Addressing this gap is essential not only for evidence‐based policy and intervention development, but also for promoting culturally sensitive and regionally relevant approaches to improving student mental health outcomes. Therefore, the aim of this study was to evaluate the mental health of dental students in Arabic‐speaking countries namely, Saudi Arabia, United Arab Emirates, Yemen and Egypt.

## 2 Methods

### 2.1 Research Ethics

This study was conducted in compliance with the ethical principles outlined in the declaration of Helsinki for research involving humans, including research on identifiable human material and data. Ethical approval was obtained from the Deanship of Scientific Research, XX (Approval Number: KFU‐REC‐2023‐OCT‐ETHICS1297 dated 10/04/2023). Participation was voluntary and confidential/anonymous. All participating students were required to provide their consent before providing their responses to an online survey.

### 2.2 Study Design and Settings

This research was an analytical cross‐sectional study based on an online survey using google forms. Data was collected from 20th May to 20th July 2024.

### 2.3 Sample Size Calculation

Power analysis with G*Power software (version 3.1) was used for sample size calculation [15]. In the Chi‐squared analyses reported, with the degrees of freedom ranging from 8 and 12, and using *α* = 0.05, the total sample size required to maintain a power of 0.90 and detect small‐to‐medium effects (*w* = 0.2) was estimated to be between *n* = 386 and *n* = 546. These are also sufficient to detect small effect sizes using analyses such as *t*‐test to compare differences in mean scores between independent groups.

### 2.4 Sampling Technique and Participants

A non‐probability convenience sampling technique was used to target undergraduate dental students enrolled at dental institutions in Saudi Arabia, Yemen, United Arab Emirates and Egypt. The selection of the participating institutions was based on the professional network of the research team and the willingness of eligible institutions to collaborate in the research. Students who had interrupted their studies and those under 18 years were excluded.

The target participants were invited to participate in an online survey through their faculty representatives. The faculty representatives shared the invitation to participate with students in all years of the undergraduate dental programme at their respective institutions through the students' affairs. A reminder was sent 2 weeks later.

### 2.5 Data Collection Instruments

The survey questionnaire consisted of five sections and was administered online using google forms (Full questionnaire is attached as an Appendix A).

The first section was related to informed consent from participants confirming their participation was voluntary and that they understood the purpose and scope of the study and that all data related to the study will be processed anonymously. The second section was related to demographic information of the participants including age, gender, institution, year of study and financial support. The third section was based on a nine‐item version of the Patient Health Questionnaire (PHQ‐9) which is a validated, and widely used instrument used to screen for depression [16]. The fourth section included a 21‐item Depression, Anxiety and Stress Scale (DASS‐21). DASS‐21 is also a validated and widely used instrument to screen for mental health issues [17]. Each of the three subscales within DASS‐21 consists of 7 items scored on a Likert scale from 0 to 3. The scoring structure and cut‐off points for PHQ‐9 and DASS‐21 subscales are elaborated further in the results section. The final section consisted of two open‐ended items regarding any reported underlying factors which may be responsible for adverse effects on participants' mental health and recommendations for improving support for students experiencing mental health issues.

### 2.6 Data Analysis

Data was analysed using the R statistical environment for Windows (R Core Team, 2022) [18]. Descriptive statistics were used to describe the sample and subgroups, and their distributions of scores for each scale, and between levels of each factor.

PHQ‐9 responses to each item were scored as follows: Not at all = 0, Several days = 1, More than half the days = 2, and Nearly every day = 3. Item scores were summed across the nine items to provide an overall PHQ‐9 score, and severity of depression was categorised as follows:: None (0–4), Mild (5–9), Moderate (10–14), Moderately Severe (15–19), and Severe (20–27).

DASS‐21 responses to each item were scored as follows: Did not apply to me at all = 0, Applied to me to some degree, or some of the time = 1, Applied to me to a considerable degree or a good part of time = 2, Applied to me very much or most of the time = 3. Items scores were then summed to give an overall DASS‐21 score, and groups of items summed to provide Depression (Items 3, 5, 10, 13, 16, 17, 21), Anxiety (Items 2, 4, 7, 9, 15, 19, 20), and Stress (Items 1, 6, 8, 11, 12, 14, 18) subscale scores. The scores of participants for each subscale were used to categorise the severity of each dimension based on thresholds shown below:

- *Depression*: Normal (0–4); Mild (5–6); Moderate (7–10); Severe (11–13); and Extremely severe (14+)
- *Anxiety*: Normal (0–3); Mild (4–5); Moderate (6–7); Severe (8–9); and Extremely severe (10+)
- *Stress*: Normal (0–7); Mild (8–9); Moderate (10–12); Severe (13–16); and Extremely severe (17+)

Chi‐squared tests of association were computed to compare the distribution of severity categories between groups on each scale. Pearson's correlation coefficients were used to evaluate relationships between scale scores.

## 3 Results

### 3.1 Demographics

Of the 508 responses received, 429 provided complete PHQ‐9 and DASS‐21 scales, and these form the basis of the analyses; participants with any missing PHQ‐9 or DASS‐21 item responses were excluded as thresholds are based on summation across items and scaling scores to account for missing data might have resulted in inaccuracies.

Out of 429 complete responses, 61.77% (*n* = 265) were from female participants, 38.23% (*n* = 164) from male participants. The modal age group was 22–25‐year‐olds (59.44%, *n* = 255), followed by 18–21‐year‐olds (33.10%, *n* = 142), with a minority being older (5.59%, *n* = 24, at 26–29; 1.86%, *n* = 8 at 30 or more).

All stages of study were represented; Year 1, 4.66%, *n* = 20; Year 2, 9.32%, *n* = 40; Year 3, 13.29%, *n* = 57; Year 4, 27.51%, *n* = 118; Year 5, 34.73%, *n* = 149; and Year 6, 10.49%, *n* = 45.

Responses were received from multiple institutions across four countries including Saudi Arabia (Jazan University, Imam Abdulrahman bin Faisal University, Majmaah University, Vision Colleges–Riyadh), Yemen (Sana'a University of Science and Technology, Ibb University, Taiz University, Ibn Al‐Nafis University, Yamenia University), UAE (Ajman University), and Egypt (Alexandria University, College of Dentistry Arab Academy of Science and Technology). Details of participants' institutions are provided Table 1.

**TABLE 1:** Institution characteristics.

Country			Financial support			Institution type		
	*n*	%		*n*	%		*N*	%
Yemen	236	55.01	Self‐Finance	266	62.00	Private	220	51.28
Saudi	103	24.01	Other	72	16.78	Public	209	48.72
Egypt	67	15.62	Sponsorship	43	10.02			
UAE	23	5.36	Scholarship	42	9.79			
			Not Specified	6	1.40			

### 3.2 Descriptive Statistics

Descriptive statistics for each scale score are shown in Table 2. The numbers of respondents in each category are shown in Table 3.

**TABLE 2:** PHQ‐9 and DASS‐21 descriptive statistics by scale and subscale.

Statistic	PHQ‐9	DASS‐21			
	Total	Total	Depression	Anxiety	Stress
Mean	11.72	23.03	7.46	6.91	8.66
SD	5.76	13.75	5.28	4.92	4.90
Min	0	0	0	0	0
Max	27	63	21	21	21
Range	27	63	21	21	21
IQR	8	18	8	7	7

**TABLE 3:** Categorisation by scale and subscale.

Category	Frequency				Percentage of respondents			
	PHQ9	DASS‐21			PHQ9	DASS‐21		
		Depression	Anxiety	Stress		Depression	Anxiety	Stress
None	49	—	—	—	11.42	—	—	—
Normal	—	135	121	195	—	31.47	28.21	45.45
Mild	102	67	73	58	23.78	15.62	17.02	13.52
Moderate	147	117	67	81	34.27	27.27	15.62	18.88
Moderately Severe	85	—	—	—	19.81	—	—	—
Severe	63	67	72	62	9.86	10.49	11.27	9.70
Extremely Severe	—	95	151	43	—	14.87	23.63	6.73

PHQ‐9, DASS‐21 Depression, Anxiety, and Stress scores were all significantly positively correlated for the whole sample, and within each subgroup of each factor (within Males, Females, 18–21‐year‐olds, 22–25‐year‐olds, Private, Public, and Other institution types, Self‐ and Other‐financed respondents). Whole‐sample correlation coefficients are shown in Table 4.

**TABLE 4:** Pearson correlation coefficients (*r*) for correlations between each scale.^a^

		DASS‐21		
		Depression	Anxiety	Stress
PHQ‐9 Total		0.73	0.65	0.70
DASS21	Depression	—	0.72	0.76
	Anxiety	—	—	0.75

^a^ Values based on all complete responses. All values are statistically significant at *p* < 0.001.

### 3.3 PHQ‐9

#### 3.3.1 Gender

**FIGURE 1:**
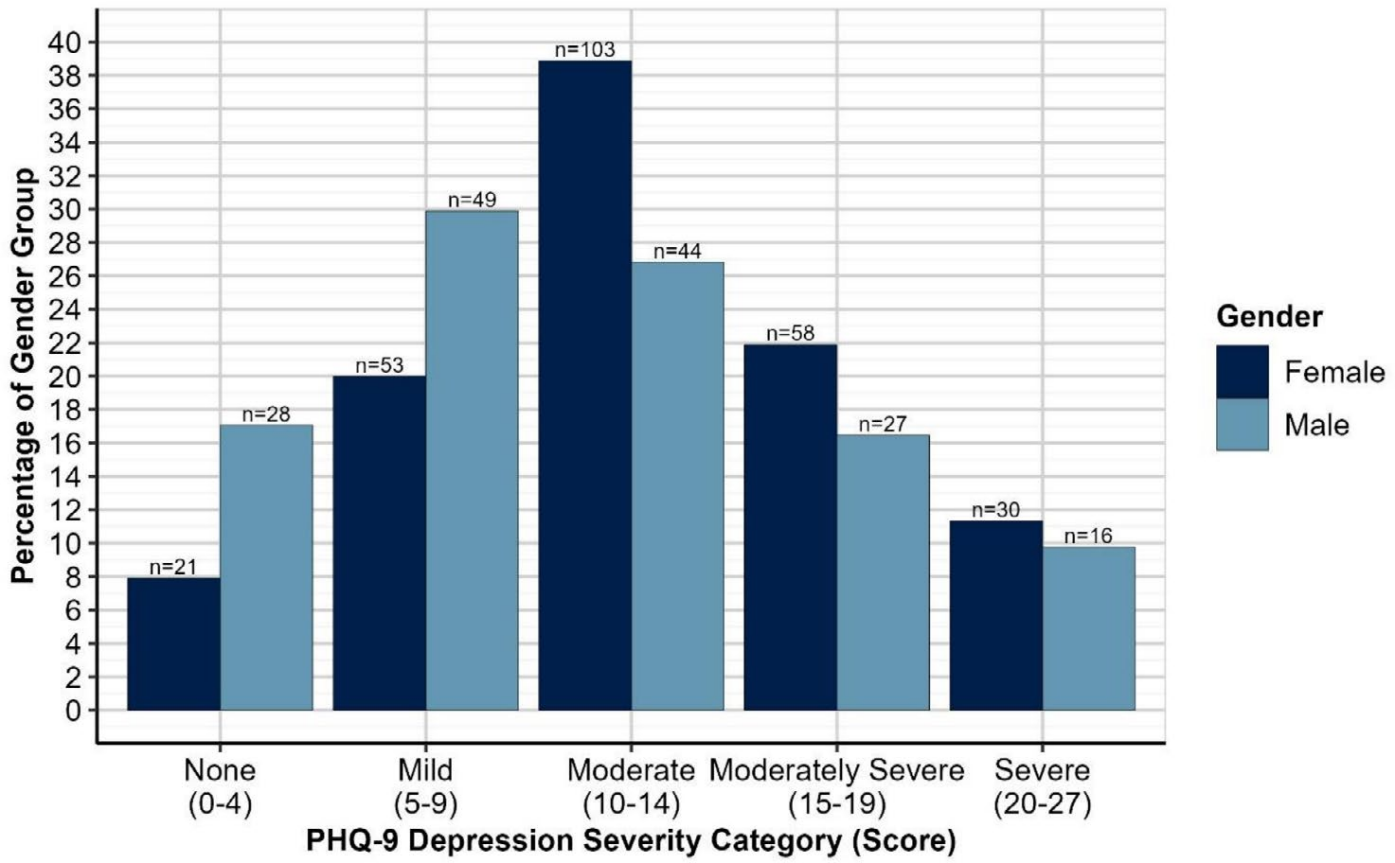
PHQ‐9 depression severity category by gender.

Female respondents scored significantly higher on the PHQ‐9 (*M* = 12.45, SD = 5.44) than male respondents (*M* = 10.45, SD = 6.08; *t*(316.46) = 3.28, *p* = 0.001). The distribution of depression severity categorizations also differed significantly between genders; *Χ* ^2^ (4) = 17.60, *p* = 0.001 (Figure 1).

#### 3.3.2 Year of Study

**FIGURE 2:**
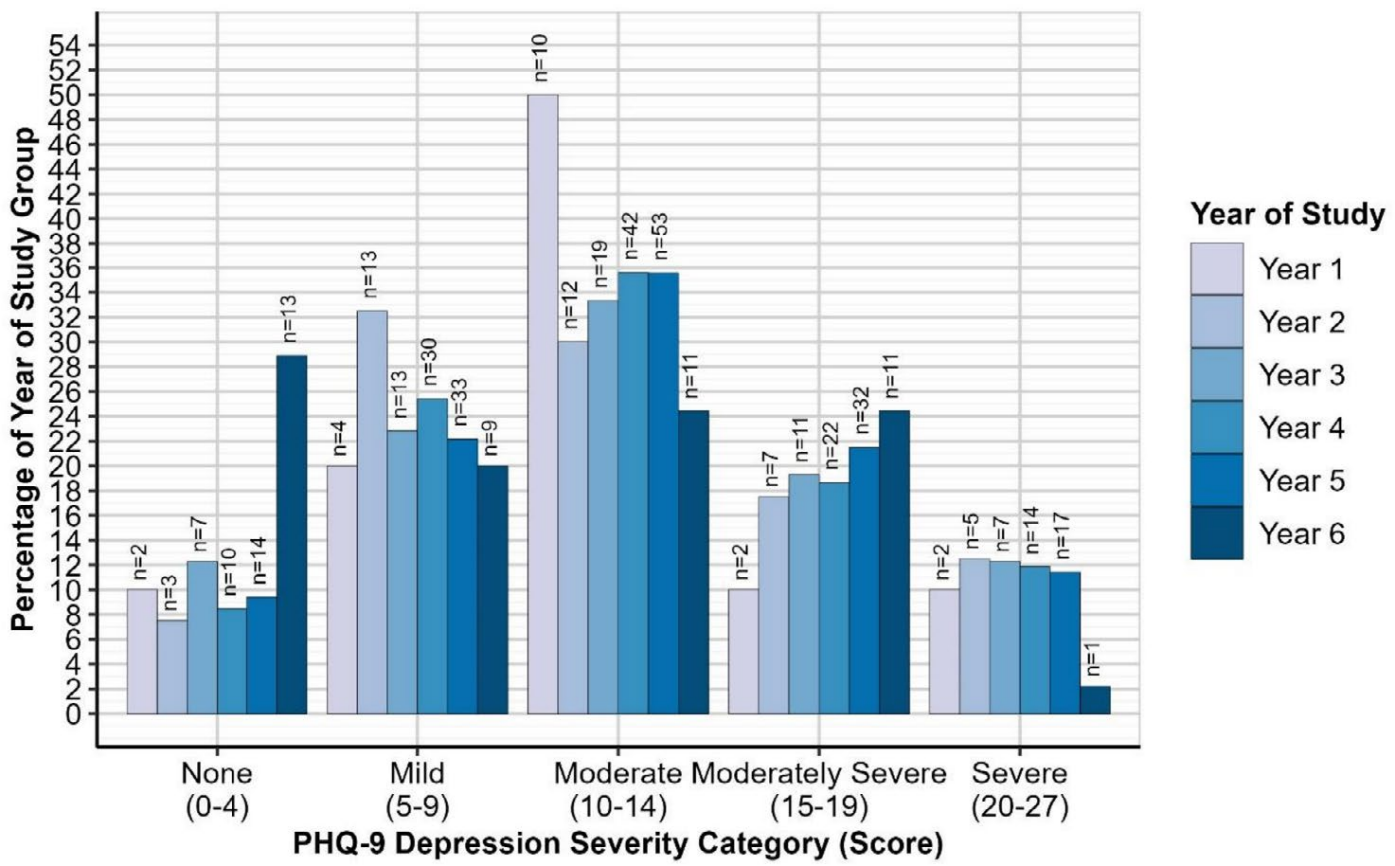
PHQ‐9 depression severity category by year of study.

Year of Study was found to have no overall main effect on PHQ‐9 scores; *F*(5,423) = 1.35, *p* = 0.244. Variations in PHQ‐9 scores are depicted in Figure 2. As with the main effect analysis, no pairwise comparisons (Tukey HSD) or associations (Chi‐Squared) were found to be statistically significant.

#### 3.3.3 Country

Country showed no association with PHQ‐9 depression severity category; *Χ* ^2^ (12) = 11.98, *p* = 0.447.

#### 3.3.4 Financial Support

The financial support status of respondents, when considered in terms of Self‐Financed vs. Other (sponsorship, scholarship, some other form of financial support status, or not specified) showed no association with PHQ‐9 depression severity category; *Χ* ^2^ (4) = 1.77, *p* = 0.777. No effect association was found when considering all categories separately either; *Χ* ^2^ (16) = 20.56, *p* = 0.196.

#### 3.3.5 Institution Type

Institution Type (Public, Private) showed no association with PHQ‐9 depression severity category; *Χ* ^2^ (4) = 3.44, *p* = 0.488.

#### 3.3.6 Age Group

Age Group was not associated with PHQ‐9 depression severity categories; *Χ* ^2^ (4) = 1.65, *p* = 0.800. Other Age Groups were excluded from this analysis due to the small numbers in each category.

### 3.4 DASS‐21

#### 3.4.1 Gender

**FIGURE 3:**
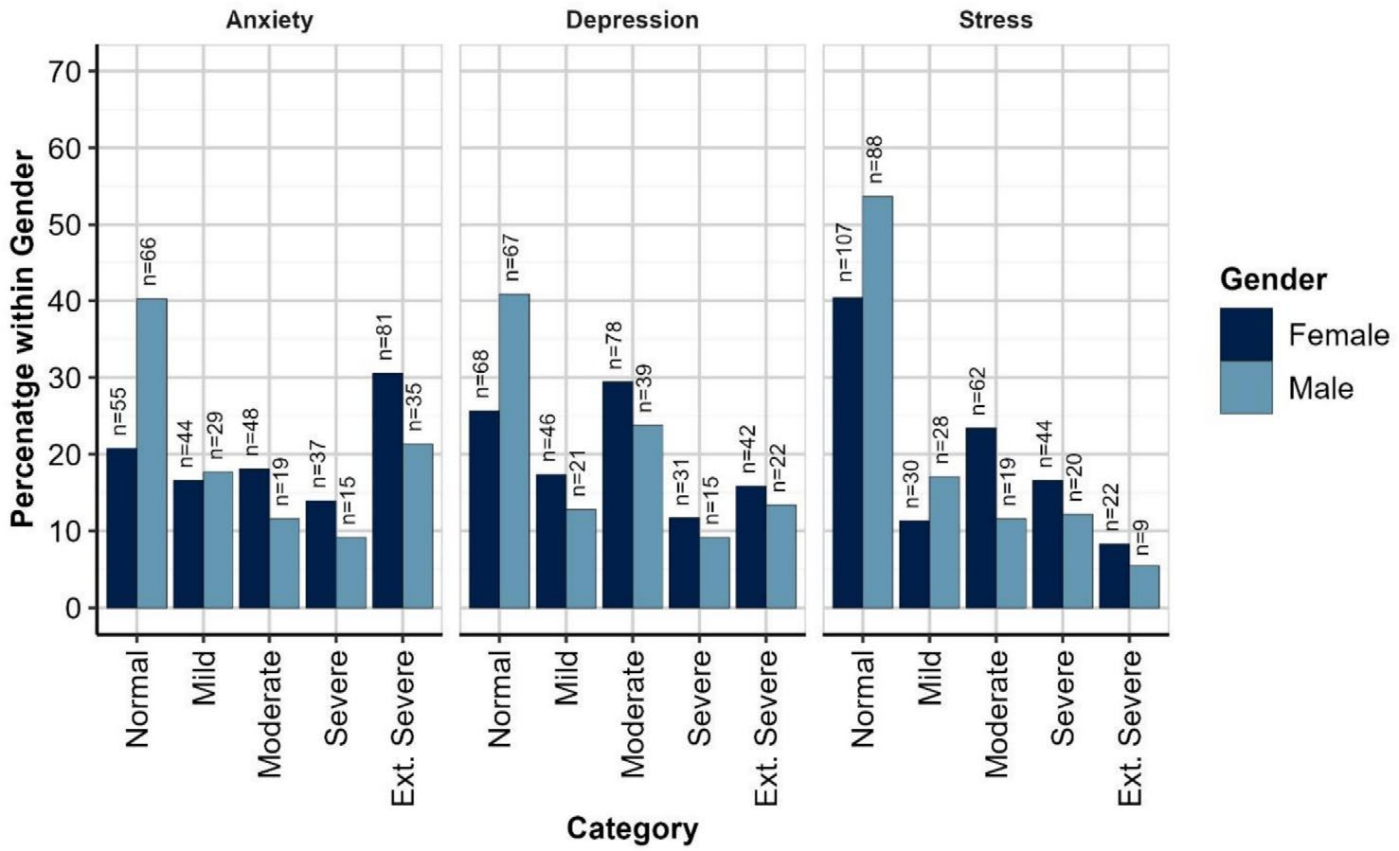
DASS‐21 subscale severity category by gender.

Gender was associated with severity category for each of the DASS‐21 subscales: Depression (*Χ* ^2^ (4) = 10.98, *p* = 0.027), Anxiety (*Χ* ^2^ (4) = 21.60, *p* < 0.001), and Stress (*Χ* ^2^ (4) = 16.33, *p* = 0.003); distributions are shown in Figure 3 which suggest possibly larger numbers of more severe categorizations in female than male respondents.

#### 3.4.2 Year of Study

**FIGURE 4:**
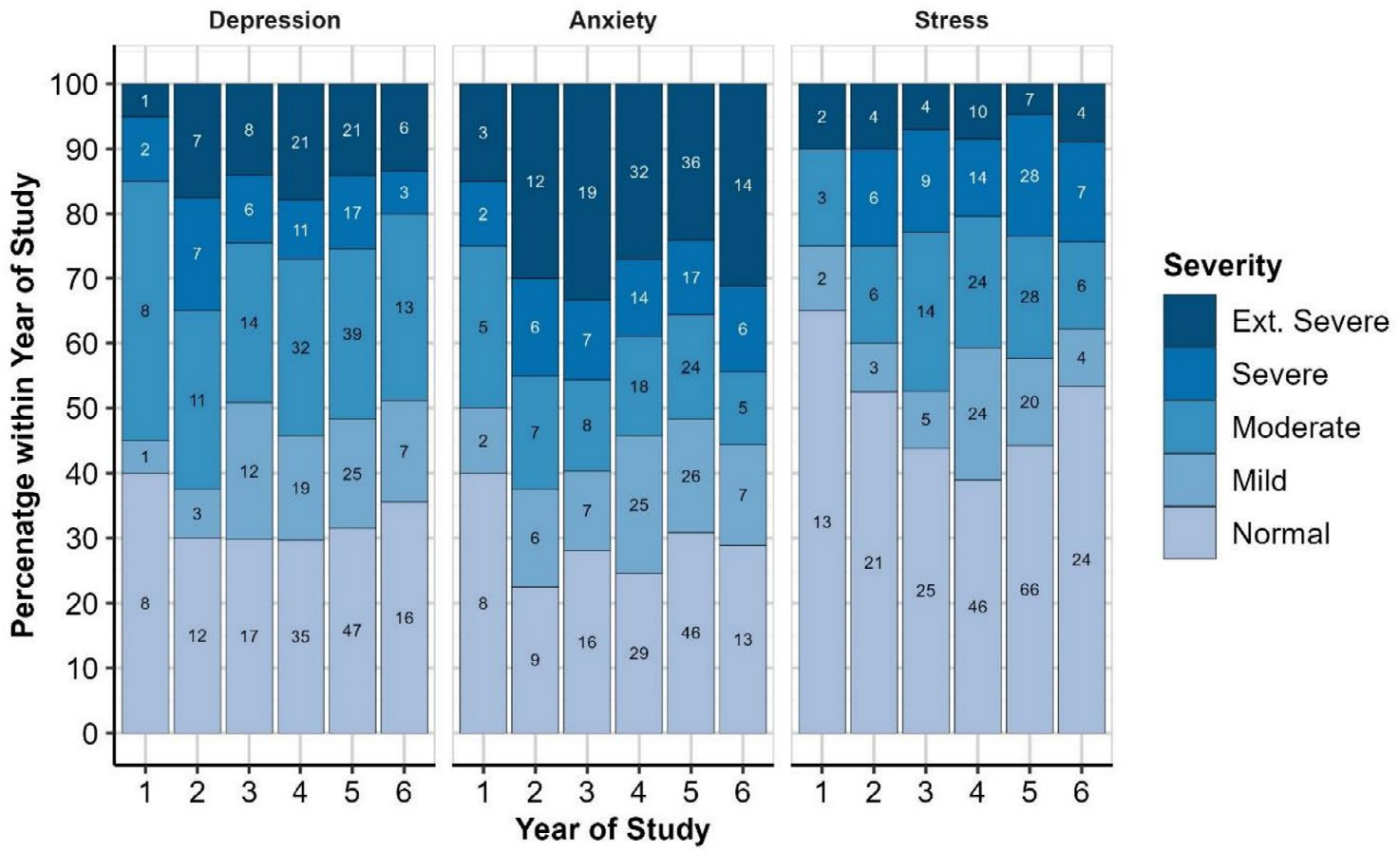
DASS‐21 subscale severity by year of study. Values within each bar indicate the frequency of respondents in that category.

Year of Study was not associated with severity of DASS‐21 Depression (*Χ* ^2^ (20) = 11.73, *p* = 0.925), Anxiety (*Χ* ^2^ (20) = 10.26, *p* = 0.963), or Stress (*Χ* ^2^ (20) = 20.84, *p* = 0.406) categories. Severity category distributions for each subscale by Year of Study are shown in Figure 4.

#### 3.4.3 Country

Country showed no association with DASS‐21 Depression (*Χ* ^2^ (12) = 17.63, *p* = 0.127), Anxiety (*Χ* ^2^ (12) = 19.56, *p* = 0.076), or Stress (*Χ* ^2^ (12) = 16.52, *p* = 0.169) severity categories.

#### 3.4.4 Financial Support

The financial support status of respondents, when considered in terms of Self‐Financed vs. Other (Sponsorship, Scholarship, some other form of financial support status, or not specified) showed no association with DASS‐21 Depression (*Χ* ^2^ (4) = 9.20, *p* = 0.056), Anxiety (*Χ* ^2^ (4) = 2.73, *p* = 0.604), or Stress (*Χ* ^2^ (4) = 1.07, *p* = 0.899) severity categories.

#### 3.4.5 Institution Type

Institution Type (Public, Private) showed no association with DASS‐21 Depression (*Χ* ^2^ (4) = 5.30, *p* = 0.258), Anxiety (*Χ* ^2^ (4) = 3.21, *p* = 0.523), or Stress (*Χ* ^2^ (4) = 4.27, *p* = 0.370) severity categories.

#### 3.4.6 Age Group

**FIGURE 5:**
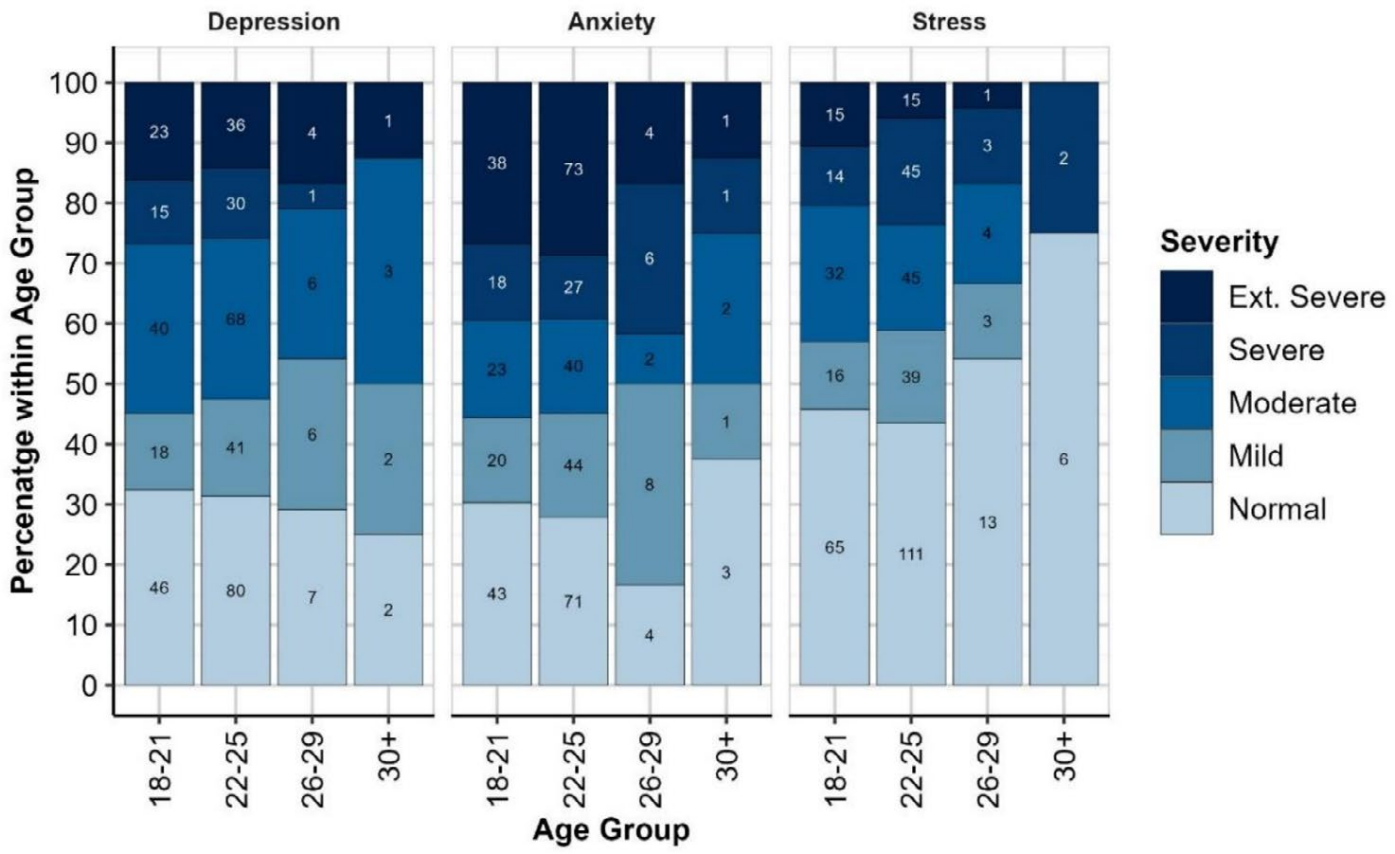
DASS‐21 subscale severity by age group. Values within each bar indicate the frequency of respondents in that category.

Age Group was not associated with DASS‐21 Depression (*Χ* ^2^ (4) = 1.20, *p* = 0.879), Anxiety (*Χ* ^2^ (4) = 1.24, *p* = 0.872), or Stress (*Χ* ^2^ (4) = 8.66, *p* = 0.070) severity categories. As with PHQ‐9 Age Group analyses, the 26–29 and 30+ Age Groups have been excluded from these analyses due to small numbers in each category. Severity category distributions for each subscale by Age Group are shown in Figure 5.

## 4 Discussion

The present study aimed to explore the mental health challenges faced by undergraduate dental students across multiple institutions in four Middle Eastern countries including Saudi Arabia, Yemen, the United Arab Emirates, and Egypt. The results highlight the significant prevalence of mental health issues, including depression, anxiety, and stress, among these students. These findings align with existing literature on mental health among medical and dental students, emphasizing the urgent need for targeted interventions and support systems within dental education programs.

### 4.1 Prevalence of Mental Health Issues

The study revealed that a substantial proportion of dental students experience varying levels of depression, anxiety, and stress. Specifically, according to the PHQ‐9 scale, 34.27% of students self‐reported moderate depression, while 9.86% reported severe depression. Measured by the DASS‐21 scale, 27.27% of respondents self‐reported moderate depression, while 14.87% reported extremely severe depression. These findings are consistent with previous studies that have documented high rates of psychological disorders among healthcare students [19]. For instance, a study conducted in Brazil found that 30.6% of medical students experienced depressive symptoms, while 32.9% suffered from anxiety [20]. Similarly, research conducted in Hong Kong among nursing students indicated that 37.3% experienced anxiety and 35.7% suffered from depression [21]. These results suggest that mental health issues are prevalent among healthcare students worldwide, and dental students are not exempt from this trend.

A study conducted among pre‐clinical dental students in Pakistan reported that 87% of students had extremely severe anxiety levels, 45% had extremely severe depression, and 31% experienced extreme levels of stress [22]. Similarly, another study in Malaysia found that the prevalence of depression, anxiety, and stress among dental students was 60.4%, 75.2%, and 50.4%, respectively [23]. These findings indicate that dental students face significant psychological challenges, with academic pressures being a key contributing factor. Recent studies from the Middle East highlight significant mental health concerns among dental students. For example, a study conducted at Jazan University in Saudi Arabia revealed high levels of depression, anxiety, and stress among healthcare students, particularly dental students. Female students were found to be at higher risk for anxiety and depression, with academic burdens identified as a primary stress trigger [24].

Several studies have highlighted the unique stressors faced by dental students, which may contribute to the high prevalence of mental health issues observed in this population [25, 26]. The demanding nature of dental curricula, which includes rigorous clinical requirements, patient management, and academic pressure, has been identified as a significant source of stress [25, 26]. The current study's findings reinforce these observations, with many students reporting academic workload and fear of failure as major contributors to their mental health challenges. This is consistent with findings from studies in other regions. For example, a study in the United Kingdom reported that medical and dental students often face stress related to the intensity and volume of their curriculum, leading to emotional and physical exhaustion [27].

When comparing the mental health of dental students with their medical counterparts, it is noteworthy that dental students often report higher levels of stress. A study conducted by Silverstein and Kritz‐Silverstein found that dental students experienced significantly higher stress levels compared to medical students, which were attributed to the unique challenges of dental education, such as the pressure to develop fine motor skills and manage patient anxiety [10]. Similarly, a study in Turkey reported that dental students faced considerable stress due to their academic responsibilities and the perceived lack of time for relaxation and self‐care [28].

### 4.2 Gender Differences

The present study also found significant gender differences in the prevalence of depression and anxiety, with female students reporting higher levels of both compared to their male counterparts. It is noteworthy that the number of female participants was higher in the study sample which may have potentially affected the results. Nevertheless, pairwise comparisons (Tukey HSD) and associations (Chi‐Squared) were computed to analyze the impact of gender on participant scores. Moreover, the gender distribution of the participants reflects the population of dental schools in most parts of the world as there are a higher number of females [24]. Studies have consistently shown that female healthcare students are more likely to experience higher levels of stress, anxiety, and depression than males [29]. This may be due to a combination of factors, including societal expectations, gender roles, and differences in coping mechanisms. Research suggests that female dental students may be more likely to internalize stress, leading to higher rates of anxiety and depression [30, 31, 32].

### 4.3 Impact of Year of Study

While some studies have suggested that senior students, particularly those in clinical years, may experience higher levels of stress due to increased academic and clinical responsibilities [33, 34], the current study did not find a significant correlation between the year of study and mental health outcomes. This is in line with several studies showing that the year of study may not always be a significant factor in determining mental health outcomes for dental students [35, 36, 37]. Resilience has been reported to play a more crucial role in mental health, with the year of study showing no significant impact once resilience was accounted for [38]. Resilience is defined as the ability to “bounce back” or recover from stressors, which plays a crucial role in shaping the mental health of dental students. It is a protective factor that helps students manage the significant psychological and occupational stress inherent in dental education [35]. Building resilience involves fostering coping strategies, self‐esteem, and adaptive responses to adversity, which can be cultivated through curricular activities aimed at promoting mental well‐being. Resilience is positively associated with better mental health outcomes, including reduced stress and improved psychological well‐being [36, 38]. Moreover, resilience is shown to mitigate common mental health disorders, such as depression and anxiety, which are prevalent among dental students [36].

Other studies have also reported no significant relationship between the year of study and mental health outcomes in their study of dental students, suggesting that stress and mental health challenges may be pervasive across all academic years [37]. No significant differences in psychiatric disorders between first‐ and final‐year dental students were reported by another study [39].

### 4.4 Financial Support

In terms of financial support status of respondents, the current study found that when considering the self‐financed vs. other status (sponsorship, scholarship, some other form of financial support status, or not specified) of participants, results revealed no association with DASS‐21 depression, anxiety, or stress severity categories, nor with the PHQ‐9 depression severity category. Several studies have also explored the relationship between financial support status and depression severity, with findings indicating that the type of financial support, such as being self‐financed or receiving sponsorship, scholarship, or other forms of financial aid, does not significantly correlate with the severity of depression as measured by the PHQ‐9 [40, 41].

Elaborating on this relationship, research suggests that while the financial burden can contribute to overall anxiety and stress levels, it does not necessarily translate into higher depression severity when controlling for other factors [41, 42, 43]. For example, students with adequate financial resources might still experience significant academic and social pressures that contribute to depression, while those with financial difficulties may have strong support networks or coping mechanisms that mitigate the impact on their mental health [16, 41]. On the other hand, financial difficulties, such as high tuition costs and living expenses, can exacerbate stress levels, leading to feelings of anxiety, hopelessness, and depression among students [42]. This financial burden often intersects with the already demanding nature of dental education, intensifying the risk of burnout and mental health deterioration [26]. Furthermore, students with insufficient financial resources may experience a sense of inequity or inadequacy compared to their peers, which can further contribute to depressive symptoms [42].

This complexity highlights that the relationship between financial status and mental health is multifaceted, with individual resilience, social support, and access to mental health resources playing critical roles in mediating the effects of financial stress on depression and anxiety symptoms [43, 44].

### 4.5 Country and Institutional Differences

The study also explored the impact of country and institutional type on mental health outcomes. No significant difference was found among different countries explored within the Middle East. For both instruments (PHQ‐9 and DASS), results showed no association with depression, anxiety, or stress severity categories. Previous studies have suggested that country may not have a significant influence on the mental health outcomes of students. For example, McKenzie et al. (2022) found that mental health outcomes among dental students were primarily influenced by individual resilience rather than country or ethnic background [38]. Similarly, Maragha et al. (2023) reported that nationality did not appear to play a decisive role in the mental health or stress levels of dental students [35]. These findings suggest that mental health challenges in dental education are often universal and not strongly influenced by the student's nationality or country of residence. However, this contrasts with another study highlighting cultural differences in the perception and stigma of mental health issues, which may influence help‐seeking behaviours and reported levels of anxiety and depression [45].

Furthermore, the lack of association between institutional type (public vs. private) and mental health outcomes suggests that the challenges faced by dental students are pervasive across different educational settings. Recent studies have also reported that there is no significant association between the type of health institution, whether public or private, and mental health outcomes among undergraduate students [46]. Despite variations in resources and academic environments, mental health challenges encountered by dental students, including stress, burnout, and anxiety, are consistent across different educational settings [19]. These challenges are influenced by the inherent demands of dental education [47], such as high academic pressure, clinical workload, and the balance between theoretical and practical training [26, 46]. Institutional factors alone do not sufficiently explain mental health issues in dental students, underscoring the need for targeted support strategies that address the common stressors present in both public and private institutions.

### 4.6 Implications for Practice

Findings of this study have several important implications for policy and practice. First, the high prevalence of mental health issues among dental students is alarming. Dental students represent a core component of the future healthcare workforce and poor mental health is likely to have an adverse effect on the quality of dental services offered to the community. The findings of this study underscore the need for targeted mental health interventions within dental schools. These could include, but are not limited to stress management workshops, counselling services, and peer support programs designed to help students cope with the unique challenges of dental education.

Additionally, the significant gender differences observed in this study suggest that interventions may need to be tailored to address the specific needs of female students, who may be at greater risk of anxiety and depression. Educational institutions could consider implementing gender‐sensitive mental health programs that address the particular challenges faced by vulnerable students.

Moreover, the study's findings related to the impact of nationality shed light on the need for culturally sensitive mental health services that take into account the diverse backgrounds of students and tackle stigma. Institutions should ensure that mental health support is accessible and acceptable to all students, regardless of their cultural or national background. Finally, the lack of association between the year of study and mental health outcomes suggests that interventions should be offered to students at all stages of their education, rather than focusing solely on specific year groups. Early intervention may help to prevent the development of more severe mental health issues during the progression of students in their program.

### 4.7 Limitations of the Study

This study has some limitations that need to be acknowledged. The selection of the participating institutions in this study was primarily guided by a combination of professional networks of the research team and a convenience sampling approach, which is commonly employed in exploratory and early‐phase research. These institutions were selected based on their willingness to collaborate, enabling timely and feasible data collection within the study's scope and resources. Moreover, the number of responses from the participating institutions was unequal. Given that participation in this study was voluntary for the students, the research team had no control over the number of responses received from each institution. Nevertheless, it is acknowledged that these issues may limit the generalizability of findings. Improvements in methodological rigor and stratified sampling techniques are recommended for future studies on this topic. Secondly, the data were collected at the end of the academic year and overlapped with the time of the end‐of‐year examinations, It is possible that the responses of the participants may have been influenced by stress related to assessments. It may be more appropriate to conduct such studies a few months before the end‐of‐year assessments to avoid a halo effect of assessment‐related anxiety among students. While participants were informed of the general aims and scope of the study in accordance with ethical guidelines to ensure informed consent, the specific research hypotheses were not disclosed. Nevertheless, the potential for response bias due to the self‐reported nature of the measures and participants' awareness of the study's overall focus cannot be entirely ruled out and should be acknowledged as a limitation. Finally, the study only utilized questionnaires for data collection and the use of qualitative methods could have enhanced the value of the study and may have provided a deeper understanding of the factors that contribute to the poor mental health of the participants. Nevertheless, the findings of this study highlight a high prevalence of mental health issues among dental students and underscore the need for structured mental health support services.

## 5 Conclusion

In conclusion, this study highlights the significant mental health challenges faced by dental students in the participating institutions, with a relatively high prevalence of depression, anxiety, and stress reported across the sample. The findings underscore the need for targeted mental health interventions within dental education programs, with particular attention to gender and cultural differences. By addressing these challenges, educational institutions can help promote the well‐being and success of their students, ultimately leading to a healthier and more resilient future dental workforce.

## Conflicts of Interest

The authors declare no conflicts of interest.

## Data Availability

The data that support the findings of this study are available from the corresponding author upon reasonable request.

## Appendix A Study Questionnaire

### Evaluation of the Mental Health of Undergraduate Dental Students in the Middle East

This study is being undertaken by a group of Dental academics in the Middle East and the United Kingdom to evaluate the frequency and patterns of mental health issues among undergraduate dental students in the Middle East.

* Indicates required question

**Section I: Informed consent**

Your participation is completely voluntary. You are requested to confirm all statements below.

1. I am over 18 years old*

*Mark only one option*.

□ Yes

2 I understand that my involvement in this study and particular data* from this research will remain strictly confidential. Only researchers involved in the study will have access to the data.

*Mark only one option*.

□ Yes

3 I fully and freely consent to participate in the study*

*Mark only one option*.

□ Yes
□ No

**Section II: Demographics**

1. Age (Years)*

*Mark only one option*.

□ 18–21
□ 22–25
□ 26–29
□ 30 or more

2 Gender*

*Mark only one option*.

□ Male
□ Female
□ Other_________

3 Institution Name and City

_________

4 Institution Type*

*Mark only one option*.

□ Public
□ Private
□ Other

5 Year of Study*

*Mark only one option*.

□ Year 1
□ Year 2
□ Year 3
□ Year 4
□ Year 5
□ Year 6

6 Financial support

*Mark only one option*.

□ Self‐finance
□ Sponsorship
□ Scholarship
□ Other

**Section III**

Over the last 2 weeks, how often have you been bothered by any of the following problems? Please read and answer each of the following statements.

The rating scale is as follows:

- Not at all
- Several days
- More than half the days
- Nearly every day

1. Little interest or pleasure in doing things

*Mark only one option*.

□ Not at all
□ Several days
□ More than half the days
□ Nearly every day

2 Feeling down, depressed, or hopeless

*Mark only one option*.

□ Not at all
□ Several days
□ More than half the days
□ Nearly every day

3 Trouble falling or staying asleep, or sleeping too much

*Mark only one option*.

□ Not at all
□ Several days
□ More than half the days
□ Nearly every day

4 Feeling tired or having little energy

*Mark only one option*.

□ Not at all
□ Several days
□ More than half the days
□ Nearly every day

5 Poor appetite or overeating

*Mark only one option*.

□ Not at all
□ Several days
□ More than half the days
□ Nearly every day

6 Feeling bad about yourself—or that you are a failure or have let yourself or your family down

*Mark only one option*.

□ Not at all
□ Several days
□ More than half the days
□ Nearly every day

7 Trouble concentrating on things, such as reading the newspaper or watching television

*Mark only one option*.

□ Not at all
□ Several days
□ More than half the days
□ Nearly every day

8 Moving or speaking so slowly that other people could have noticed? Or the opposite– being so fidgety or restless that you have been moving around a lot more than usual.

*Mark only one option*.

□ Not at all
□ Several days
□ More than half the days
□ Nearly every day

9 Thoughts that you would be better off dead or of hurting yourself in some way

*Mark only one option*.

□ Not at all
□ Several days
□ More than half the days
□ Nearly every day

**Section IV**

Please read and answer each of the following statements indicating how much the statement applied to you over the past week. There are no right or wrong answers. Do not spend too much time on any statement.

The rating scale is as follows:

- Did not apply to me at all
- Applied to me to some degree, or some of the time
- Applied to me to a considerable degree or a good part of time
- Applied to me very much or most of the time

1. I found it hard to wind down

*Mark only one option*

□ Did not apply to me at all
□ Applied to me to some degree, or some of the time
□ Applied to me to a considerable degree or a good part of time
□ Applied to me very much or most of the time.

2 I was aware of the dryness of my mouth

*Mark only one option*.

□ Did not apply to me at all
□ Applied to me to some degree, or some of the time
□ Applied to me to a considerable degree or a good part of time
□ Applied to me very much or most of the time

3 I could not seem to experience any positive feeling at all

*Mark only one option*.

□ Did not apply to me at all
□ Applied to me to some degree, or some of the time
□ Applied to me to a considerable degree or a good part of time
□ Applied to me very much or most of the time

4 I experienced breathing difficulty (e.g., excessively rapid breathing, breathlessness in the absence of physical exertion)

*Mark only one option*.

□ Did not apply to me at all
□ Applied to me to some degree, or some of the time
□ Applied to me to a considerable degree or a good part of time
□ Applied to me very much or most of the time

5 I found it difficult to work up the initiative to do things

*Mark only one option*.

□ Did not apply to me at all
□ Applied to me to some degree, or some of the time
□ Applied to me to a considerable degree or a good part of time
□ Applied to me very much or most of the time

6 I tended to overreact to situations

*Mark only one option*.

□ Did not apply to me at all
□ Applied to me to some degree, or some of the time
□ Applied to me to a considerable degree or a good part of time
□ Applied to me very much or most of the time

7 I experienced trembling (e.g., in the hands)

*Mark only one option*

□ Did not apply to me at all
□ Applied to me to some degree, or some of the time
□ Applied to me to a considerable degree or a good part of time
□ Applied to me very much or most of the time

8 I felt that I was using a lot of nervous energy

*Mark only one option*.

□ Did not apply to me at all
□ Applied to me to some degree, or some of the time
□ Applied to me to a considerable degree or a good part of time
□ Applied to me very much or most of the time

9 I was worried about situations in which I might panic and make a fool of myself

*Mark only one option*.

□ Did not apply to me at all
□ Applied to me to some degree, or some of the time
□ Applied to me to a considerable degree or a good part of time
□ Applied to me very much or most of the time

10 I felt that I had nothing to look forward to

*Mark only one option*.

□ Did not apply to me at all
□ Applied to me to some degree, or some of the time
□ Applied to me to a considerable degree or a good part of time
□ Applied to me very much or most of the time

11 I found myself getting agitated

*Mark only one option*.

□ Did not apply to me at all
□ Applied to me to some degree, or some of the time
□ Applied to me to a considerable degree or a good part of time
□ Applied to me very much or most of the time

12 I found it difficult to relax
  *Mark only one option*.

□ Did not apply to me at all
□ Applied to me to some degree, or some of the time
□ Applied to me to a considerable degree or a good part of time
□ Applied to me very much or most of the time

13 I felt down‐hearted and blue

*Mark only one option*.

□ Did not apply to me at all
□ Applied to me to some degree, or some of the time
□ Applied to me to a considerable degree or a good part of time
□ Applied to me very much or most of the time

14 I was intolerant of anything that kept me from getting on with what I was doing

*Mark only one option*.

□ Did not apply to me at all
□ Applied to me to some degree, or some of the time
□ Applied to me to a considerable degree or a good part of time
□ Applied to me very much or most of the time

15 I felt I was close to panic

*Mark only one option*.

□ Did not apply to me at all
□ Applied to me to some degree, or some of the time
□ Applied to me to a considerable degree or a good part of time
□ Applied to me very much or most of the time

16 I was unable to become enthusiastic about anything

*Mark only one option*.

□ Did not apply to me at all
□ Applied to me to some degree, or some of the time
□ Applied to me to a considerable degree or a good part of time
□ Applied to me very much or most of the time

17 I felt I was not worth much as a person

*Mark only one option*.

□ Did not apply to me at all
□ Applied to me to some degree, or some of the time
□ Applied to me to a considerable degree or a good part of time
□ Applied to me very much or most of the time

18 I felt that I was rather touchy

*Mark only one option*.

□ Did not apply to me at all
□ Applied to me to some degree, or some of the time
□ Applied to me to a considerable degree or a good part of time
□ Applied to me very much or most of the time

19 I was aware of the action of my heart in the absence of physical exertion (e.g., sense of heart rate increase, heart missing a beat)

*Mark only one option*.

□ Did not apply to me at all
□ Applied to me to some degree, or some of the time
□ Applied to me to a considerable degree or a good part of time
□ Applied to me very much or most of the time

20 I felt scared without any good reason

*Mark only one option*.

□ Did not apply to me at all
□ Applied to me to some degree, or some of the time
□ Applied to me to a considerable degree or a good part of time
□ Applied to me very much or most of the time

21 I felt that life was meaningless

*Mark only one option*.

□ Did not apply to me at all
□ Applied to me to some degree, or some of the time
□ Applied to me to a considerable degree or a good part of time
□ Applied to me very much or most of the time

**Section V**

If you believe you are experiencing mental health issues please answer the questions below:

1. In your opinion, what are the key factors affecting your mental health adversely?

_________

2 How could your institution support you better to manage your mental health?

_________

Thank you for your participation!

This content is neither created nor endorsed by Google Forms
